# Heart on a Plate: Histological and Functional Assessment of Isolated Adult Zebrafish Hearts Maintained in Culture

**DOI:** 10.1371/journal.pone.0096771

**Published:** 2014-05-13

**Authors:** Sebastian Pieperhoff, Kathryn S. Wilson, James Baily, Kim de Mora, Sana Maqsood, Sharron Vass, Jonathan Taylor, Jorge Del-Pozo, Calum A. MacRae, John J. Mullins, Martin A. Denvir

**Affiliations:** 1 UoE/BHF Centre for Cardiovascular Science, Queen’s Medical Research Institute, The University of Edinburgh, Edinburgh, Scotland, United Kingdom; 2 School of Physics and Astronomy, University of Glasgow, Glasgow, Scotland, United Kingdom; 3 Royal Dick School of Veterinary Studies, Division of Veterinary Clinical Sciences, The University of Edinburgh, Hospital for Small Animals, Easter Bush Veterinary Centre, Roslin, Midlothian, Scotland, United Kingdom; 4 Cardiovascular Division, Brigham and Women’s Hospital, Harvard Medical School, The Broad Institute of MIT and Harvard, Boston, Massachusetts, United States of America; Heart Science Centre, Imperial College London, United Kingdom

## Abstract

The zebrafish is increasingly used for cardiovascular genetic and functional studies. We present a novel protocol to maintain and monitor whole isolated beating adult zebrafish hearts in culture for long-term experiments. Excised whole adult zebrafish hearts were transferred directly into culture dishes containing optimized L-15 Leibovitz growth medium and maintained for 5 days. Hearts were assessed daily using video-edge analysis of ventricle function using low power microscopy images. High-throughput histology techniques were used to assess changes in myocardial architecture and cell viability. Mean spontaneous Heart rate (HR, min^−1^) declined significantly between day 0 and day 1 in culture (96.7±19.5 to 45.2±8.2 min^−1^, mean±SD, p = 0.001), and thereafter declined more slowly to 27.6±7.2 min^−1^ on day 5. Ventricle wall motion amplitude (WMA) did not change until day 4 in culture (day 0, 46.7±13.0 µm vs day 4, 16.9±1.9 µm, p = 0.08). Contraction velocity (CV) declined between day 0 and day 3 (35.6±14.8 vs 15.2±5.3 µms^−1^, respectively, p = 0.012) while relaxation velocity (RV) declined quite rapidly (day 0, 72.5±11.9 vs day 1, 29.5±5.8 µms^−1^, p = 0.03). HR and WMA responded consistently to isoproterenol from day 0 to day 5 in culture while CV and RV showed less consistent responses to beta-agonist. Cellular architecture and cross-striation pattern of cardiomyocytes remained unchanged up to day 3 in culture and thereafter showed significant deterioration with loss of striation pattern, pyknotic nuclei and cell swelling. Apoptotic markers within the myocardium became increasingly frequent by day 3 in culture. Whole adult zebrafish hearts can be maintained in culture-medium for up to 3 days. However, after day-3 there is significant deterioration in ventricle function and heart rate accompanied by significant histological changes consistent with cell death and loss of cardiomyocyte cell integrity. Further studies are needed to assess whether this preparation can be optimised for longer term survival.

## Introduction

Zebrafish are increasingly used to study cardiac development, cardiac disease development, heart muscle repair and regeneration after experimental injury and in pharmacological studies and screens [1]–[5]. The fish heart consists of an inflow tract (*sinus venosus*), a single atrium and ventricle, and an outflow tract (*bulbus arteriosus*). Most fish species have a highly trabeculated ventricle, often referred to as the spongy myocardium. Very agile fish, such as migratory salmon, tuna, shark and zebrafish possess an additional thin outer shell of muscle, called the compact myocardium [6]–[10] similar to mammalian hearts in early developmental stages [11]. Despite the obvious anatomical differences, the cardiac physiology of fish and humans is similar. Action potentials of both fish and human cardiomyocytes, for example, are characterized by a long duration and a long plateau phase, different from those of mice and other rodent model systems [12], [13].

To study isolated adult mammalian and fish hearts, Langendorff working heart preparations or similar *in situ* setups have been used [14], [15]. While, such preparations allow for a broad spectrum of key biochemical, morphological and pharmacological studies to be carried out on the intact heart, they have the disadvantage of requiring relatively complex perfusion techniques. Furthermore, mammalian hearts usually cannot survive beyond 24 h *ex-vivo* [16] although interest has re-emerged in maintaining isolated rat hearts for a number of days as a method of reseeding stem-cell derived cardiomyocytes [17].

Isolated zebrafish hearts have also been studied previously focusing on measurement of twitch force generated by a single isolated heart for up to 4 hours *in* *vitro* [18]. However, we have developed an *ex vivo* adult zebrafish heart preparation that permits physiological measurements for up to 3 days in culture. This preparation allows us to monitor heart rate and ventricle function and to assess its response to beta agonist drugs. We also describe a methodology for high-throughput histological analysis of cellular changes associated with long term zebrafish heart culture.

## Materials and Methods

### Animals

Zebrafish were maintained at 28°C and fed with hard feed SDS 100–400 (Special Diet Services, UK) and brine shrimp (artemia, ZMsystems, UK). Wildtype zebrafish (WIK strain) were used for all experiments. All experiments conformed strictly to the Animals Scientific Procedures Act 1986 (United Kingdom) and were approved by the local ethical committee of the University of Edinburgh Centre for Cardiovascular Science.

### Maintenance of Adult Zebrafish Hearts in vitro

Adult zebrafish (aged 120–180 days) were euthanized by immersion in 1 M tricaine (MS222, Sigma) in PBS, until loss of righting reflexes, followed by decapitation according to local animal guidelines. Euthanized fish were immersed ventral side up on a sponge/paper trough in the bottom of a petri dish filled with excision buffer (1x PBS; 1% antibiotic/antimycotic: Penicillin, Streptomycin, Amphotericin B, Life Technologies, UK; 150 I.U./ml heparin, Fisher Scientific, UK). The hearts were carefully excised with the minimum of manipulation to avoid damage and placed in 12 well culture plates (Iwaki, UK) filled with heart culture medium (L-15 Leibovitz medium, Life Technologies; 10% FCS; 1% antibiotic/antimycotic; 1.25 mM CaCl_2_; 800 mg/l glucose). The hearts were screened for any visible abnormalities, such as mechanical trauma from excision, morphological abnormalities, cessation of heart beat or obvious arrhythmia. Only intact, rhythmically beating hearts were individually transferred to the cell culture hood (sterile) in 12-well plates. Hearts were maintained in an incubator at 28°C on a standard shaking platform (0.1–0.5 Hz) to increase oxygen perfusion for a maximum of 5 days with media changed in sterile conditions (cell culture hood) at 24 hour intervals. Aseptic technique is important for the long term maintenance of these beating zebrafish heart preparations. Using standard aseptic working techniques we rarely detected bacterial contaminations (in <0.5% of heart cultures).

### Assessment of Cardiac Function

Digital video analysis was used to assess ventricular wall motion and heart rate in cultured hearts using a technique previously validated in our lab [19].

**Figure 1:**
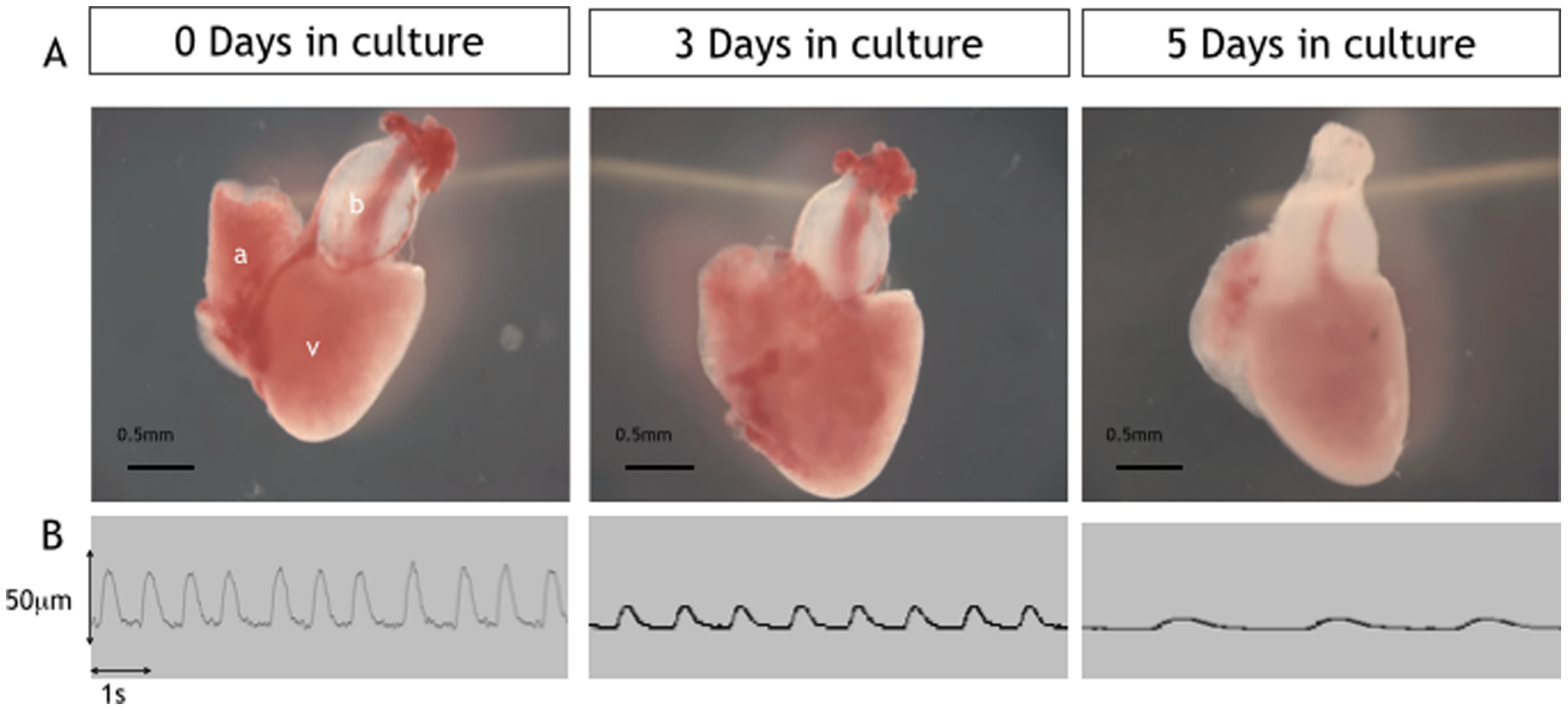
Low power images of excised cultured hearts (days 0–5). Upper panel (A) showing examples of excised hearts maintained in culture for 0, 3 and 5 days. Lower panel (B) shows example traces from Video-edge detection method displaying movement of the ventricle wall which was used to assess heart rate and ventricle function at days 0,3 and 5 in culture.

**Figure 2:**
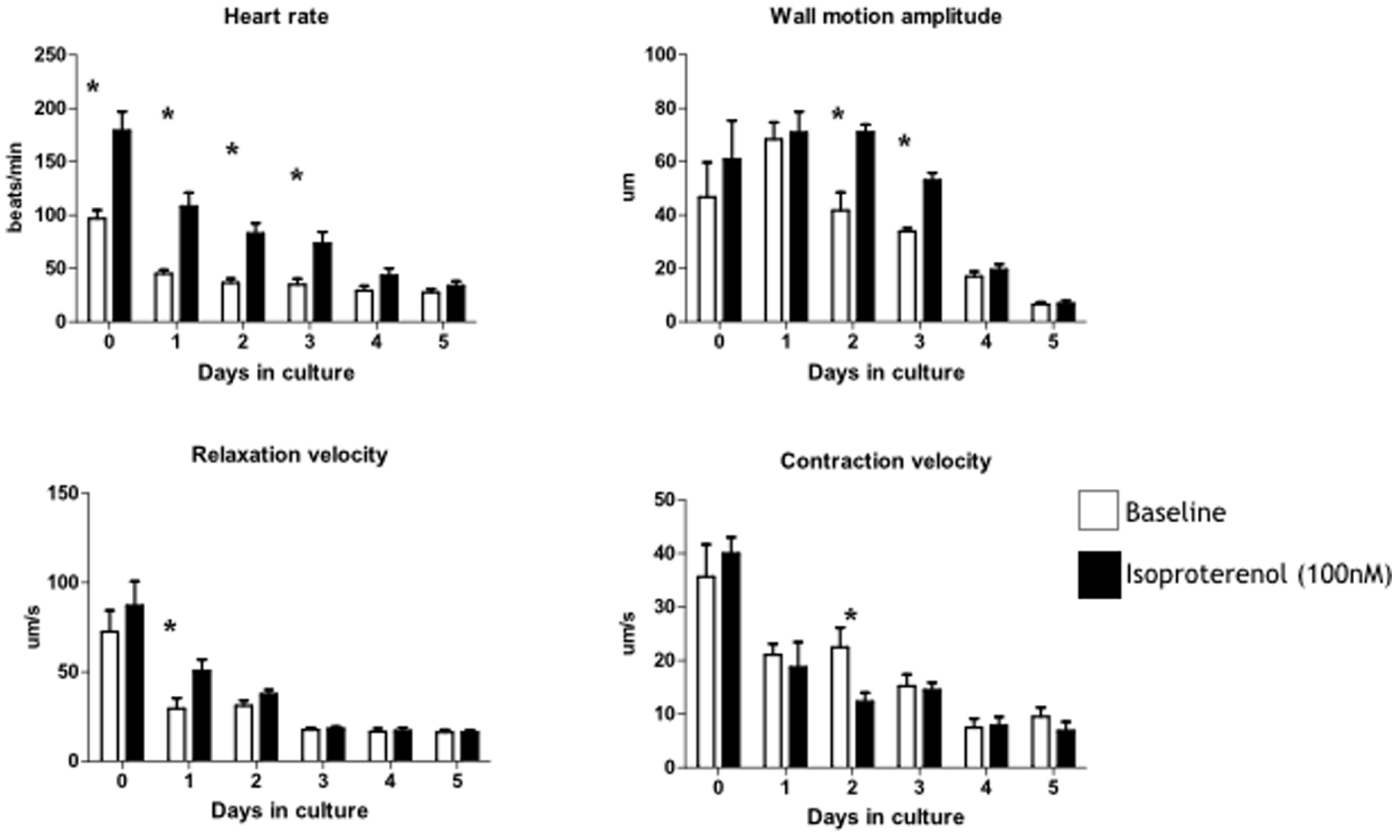
Functional assessment of isolated hearts in culture (days 0–5). Heart rate and ventricle function assessed using video edge detection of wall motion during spontaneous beating (n = 6 hearts over 5 days, mean±sem). Data analysed by 2 way ANOVA and Bonferroni correction for multiple comparison show a clear interaction of all parameters with time over the 5 days in culture and a clear interaction with response to isoproterenol (*p<0.05).

Briefly, a frame-grabber card in a computer continuously digitizes output from a video camera mounted on a low power microscope and displays it on a monitor. The user optimises the image for contrast and then selects video lines on the edge of the heart corresponding to the ventricular wall close to the apex using a small cursor. The cursor tracks the movement of the ventricle wall and displays it as a continuous trace (figure 1, panel B). The trace is calibrated using a standard graticule and then stored digitally. The trace is analysed off-line using commercial software (SoftEdge, IonOptix Corporation) for ventricle wall motion amplitude (WMA, µm), ventricle contraction velocity (CV, µm/s) and ventricle relaxation velocity (RV, µm/s) with heart rate also obtained from these traces under each experimental condition and at each time-point (figure 2).

Representative cardiac cycles over 1 minute were documented for each heart (ventricle) at baseline (day 0, within 1 hour of surgical excision) in physiological buffer (PBS) and following a switch to physiological buffer containing isoproterenol (100 nM) at the same time each day on 5 consecutive days in culture.

### Design and Manufacture of the Zebrafish Heart-mould

**Figure 3:**
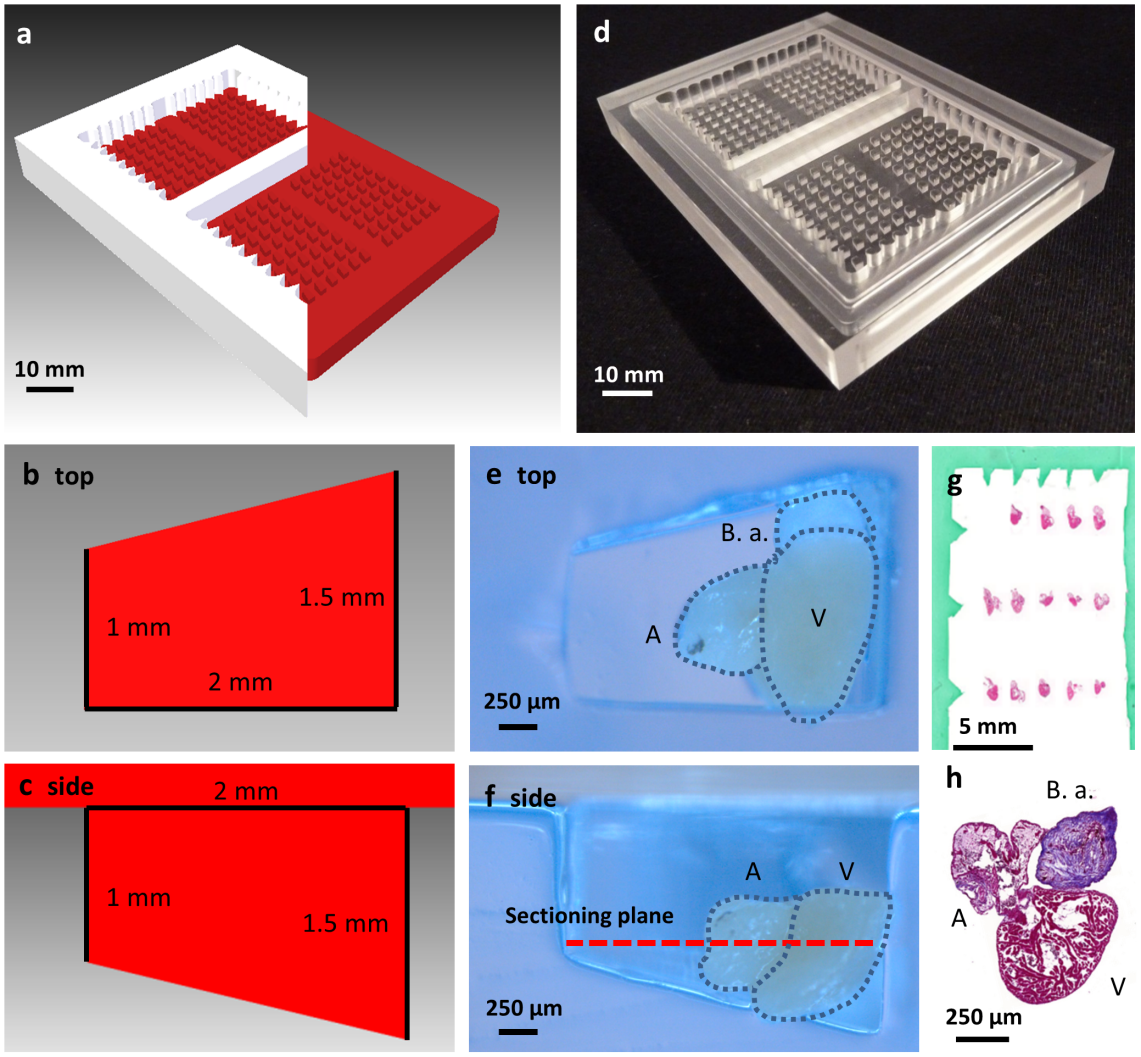
Heart mould for high throughput histology. (**a**) 3D render of cutaway top (white) and bottom part (red) of the designed heart mould. (**b**) Render of single well from above with superimposed dimensions. (**c**) 2D cutaway of a single well of the heart mould showing dimensions. (**d**) Top and bottom sections of heart mould machined from acrylic. (**e**) Top down view of single agar well with zebrafish heart. (**f**) Cut-away side view of single well showing heart and sectioning plane. (**g**) Example slide with grid of hearts with histology-dye borders showing arrow row and column markers. (**h**) High-magnification of single H&E stained heart. V, ventricle. A, atrium. B.a., *bulbus arteriosus*.

The histology heart mould, made from acrylic resin, enabled a standardized heart orientation of up to 48 hearts in a single histology cassette (Figure 3). It was engineered as a two-part structure to provide a consistent gel depth while avoiding the need to use tape to seal the inferior surface. Gel moulds were created from the acrylic mould using 1% agarose in PBS incorporating a 10% green “tissue marking dye” (CellPath, UK) in PBS to provide a series of markers thus facilitating sample tracking and location of individual hearts during and after histological processing.

### Histology and Immuno-fluorescence Microscopy

Hearts were fixed in 4% formaldehyde in PBS for 60 minutes at room temperature and subsequently 24 hours at 4°C. Samples were then washed and stored in 70% ethanol at 4°C prior embedding in paraffin wax and sectioning. We used Haematoxylin and Eosin (H&E) staining to examine cell and tissue morphology as well as Masson’s Trichrome (MT) to study deposition of collagen in the heart samples.

**Figure 6:**
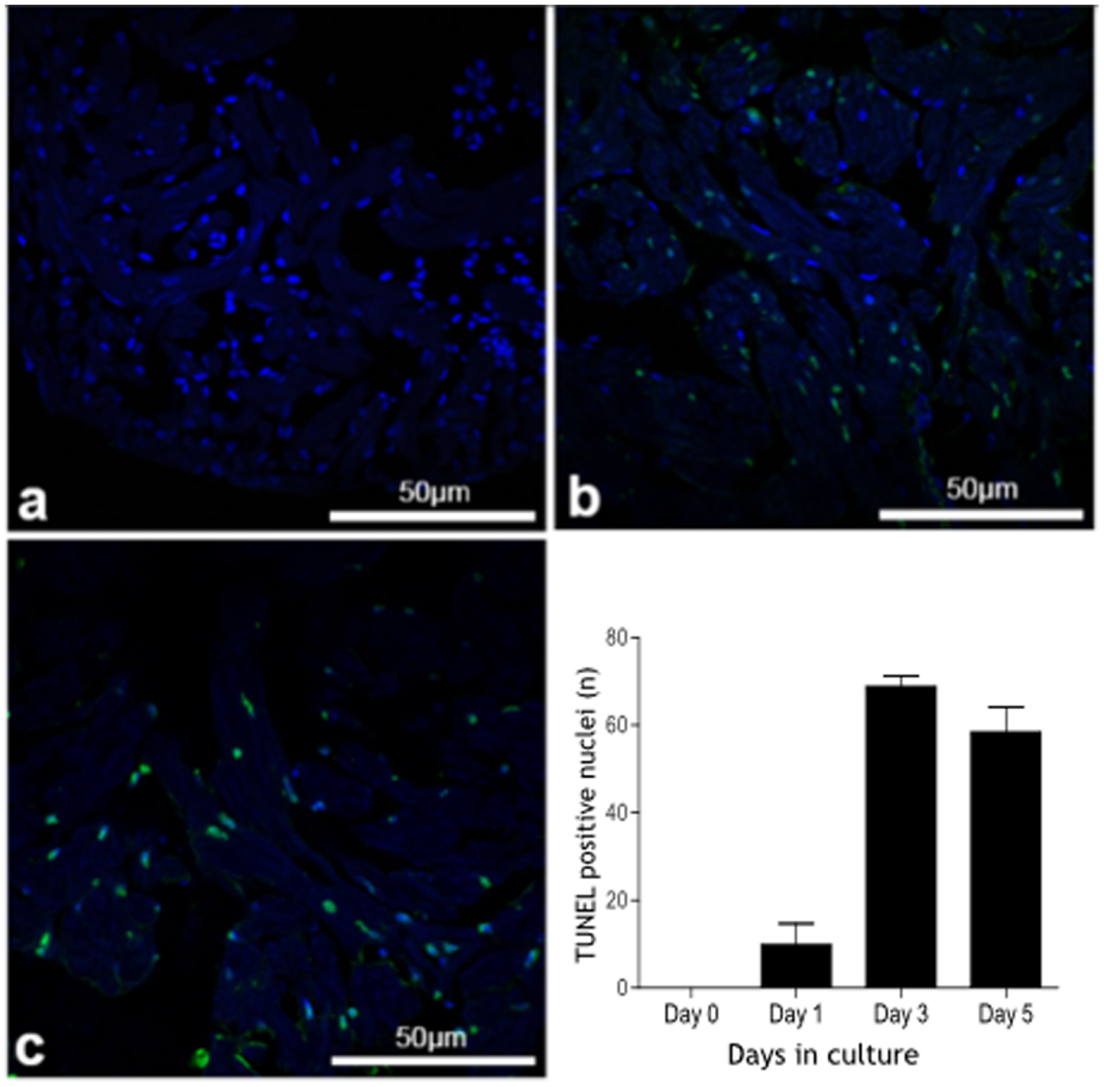
Apoptosis in isolated cultured zebrafish hearts (TUNEL staining). Apoptotic nuclei (green) and nuclear DAPI staining (blue) in zebrafish hearts (assessed by TUNEL staining) maintained in culture for 0, 1, 3 and 5 days showing a marked increase in the number of apoptotic bodies at day 3 which is maintained at day 5.

TUNEL assay (Figure 6) revealed very few apoptotic bodies on day 0 and day 1 in culture increasing markedly on day 3 in culture. This remained significantly elevated by day 5 in culture.

Samples were embedded in a pre-cast 1% agarose (in PBS) block using the “heart mould” (Figure 3) and sealed with agarose to allow for consistent orientation during throughput histology and fluorescence studies. Each heart was individually placed in a well and oriented correctly for sagittal sectioning through the ventricle, atrium and bulbus arteriosus. When all hearts had been inserted, the gel was heated to 40°C by floating on a water bath. Using a 1 ml pipette with ∼10 mm of the tip removed, agarose at ∼40–50°C was carefully added to the wells to seal the hearts in place. Excess agarose was trimmed and the block containing the hearts placed in a histology mould. Then we cast a secondary gel to create the row and column markers (see above).

For immunofluorescence microscopy, samples were cryo-preserved by snap-freezing in isopropanol, precooled in liquid nitrogen. The “snap-freeze-setup” consisted of 100 ml of isopropanol in a 200 ml polyethylene beaker in a weighted one litre polyethylene beaker surrounded by a styrofoam box containing liquid nitrogen. Hearts were placed on 10 mm×100 mm strips of Parafilm, excess culture medium was removed using delicate task wipers and OCT embedding medium (Tissue-Tek, Sakura, Japan) was added. Once the sample was oriented in the O.C.T. medium, it was submerged in the precooled isopropanol for 60–90 seconds and then placed in an Eppendorf reaction vial, precooled on dry ice. The samples were stored in low temperature resistant containers at −80°C and covered in precooled isopentane. Hearts were then sectioned on a Leica CM1900 cryomicrotome and transferred to SuperfrostPlus object slides (VWR, UK) to be air dried for 4–24 h. The sections were fixed for 15 minutes in 4% paraformaldehyde then washed for 5 minutes in PBS tween (PBST, 0.1% tween-20 in 1x PBS) followed by 10 minutes in 0.5% Triton X100 in PBS. Primary and secondary antibody incubations were performed at room temperature (RT) for 2 h each. Between antibody incubation steps slides were washed 3×5 minutes in PBS tween, followed by washing in distilled water and then in ethanol. The sections were then air-dried and embedded using Fluoromount-G (SouthernBiotech, Glasgow, Scotland, UK). We used primary antibody to sarcomeric alpha actinin (mouse, clone EA-53, Abcam), DAPI (Serva Heidelberg) and cy3-conjugated anti-mouse IgG secondary antibody (Dianova, Germany).

### Myocardial Apoptosis Assessment

Zebrafish hearts were removed from culture at day 0, 1, 3 and 5 before being fixed, embedded and sectioned as described above. 20 images at high magnification were acquired for each zebrafish heart at each time point using a Zeiss 710 confocal laser scanning microscope. DAPI positive nuclei were counted using ImageJ and compared to TUNEL positive nuclei. Four heart sections were analysed in each heart for quantification of TUNEL staining. TUNEL assay (DeadEnd™ TUNEL assay) was performed according to the suppliers protocol (Promega UK, Southampton, UK). Fluorescence microscopy was performed using a Leica LSM 710 confocal laser scanning microscope and Leica ZEN imaging software. Histological examination was performed using an Olympus BX51TF (Olympus, Japan) brightfield microscope linked to a video camera (micropublisher 3.3 RTV QImaging, Canada) and analysed using commercial software (Qcapture Pro 6.0 imaging software, QImaging).

### Statistical Analysis

Data are presented as mean ± standard deviation. Paired heart rate data were analysed using a Students paired t-test and unpaired data were analysed by 2-way ANOVA. Statistical significance was accepted at the 5% level.

## Results

Beating hearts were transferred from anaesthetised adult zebrafish into culture plates with high success rate with more than 95% surviving beyond 1 day in culture. There were gross changes in the appearance of hearts observed under low power microscopy over the course of 5 days in culture with a gradual reduction in size of the atrium and the ventricle and a gradual loss of the initial dark-red coloration of the myocardium to a paler pink (figure 1).

### Heart Function in Culture

Upon immediate placement of excised hearts into culture medium (day 0) mean heart rate (HR) was 97.8±7.9 min^−1^ (n = 6 hearts) and declined to a mean of 45.2±3.5 min^−1^ after 1 day in culture (p = 0.001, figure 2). Thereafter, HR declined more slowly to 36.5±4.0 min^−1^ by day 3 and 27.5±2.7 min^−1^ by day-5. There was a significant interaction between heart rate and days in culture (2-way ANOVA, p<0.001). HR increased in response to the beta agonist isoproterenol (100 nM) on each of days 0–3 with loss of the response by day-4 and 5 in culture. There was a significant interaction between HR and isoproterenol (2-way ANOVA, p<0.001). Ventricle wall motion amplitude (WMA) did not decline significantly until day 4 in culture (day 0, 46.7±13.0 µm vs day 4, 16.9±1.9 µm, p = 0.08). WMA did not respond significantly to isoproterenol on days 0 and 1 but showed a clear response on days 2 and 3 before being lost on days 4 and 5. There was a significant interaction between WMA and time spent in culture (p<0.001) and response to isoproterenol (p<0.01). Contraction velocity (CV) declined between day 0 and day 3 (35.6±14.8 vs 15.2±5.3 µms^−1^, respectively, p = 0.012) while relaxation velocity (RV) declined significantly within the first 24 hours in culture (day 0, 72.5±11.9 vs day 1, 29.5±5.8 µms^−1^, p = 0.03). Contraction velocity did not respond to isoproterenol throughout the 5 days in culture with the exception of day 2 where it showed a significant decline. CV and RV both showed an interaction with time spent in culture (P<0.01) but no interaction with isoproterenol (P>0.05).

### Histological Analysis of Zebrafish Hearts Grown in Culture

**Figure 4:**
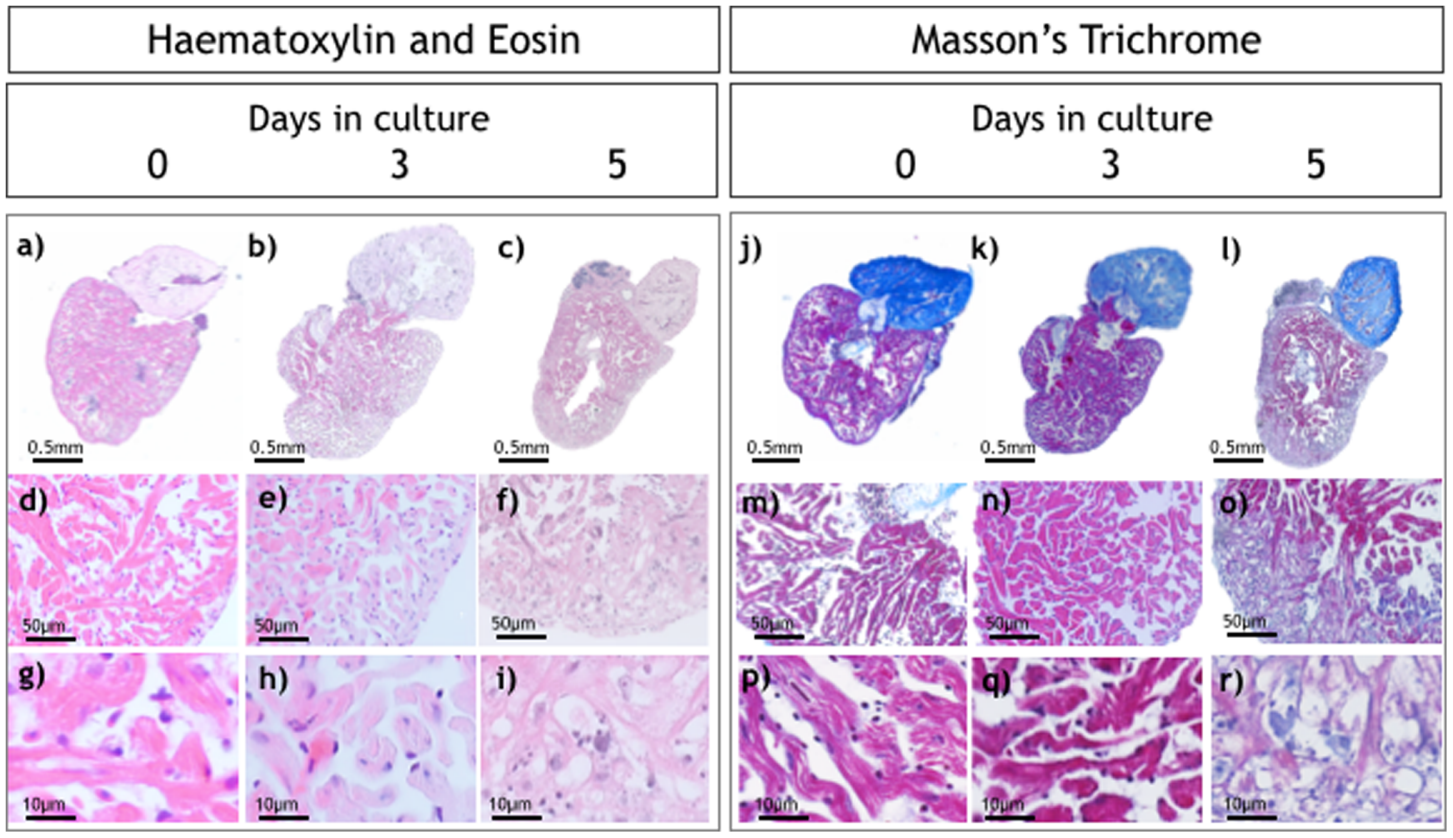
Histological analysis of isolated hearts. Haematoxylin and Eosin (H&E; a–i) and Masson’s trichrome staining (j–r) of hearts at 0, 3 and 5 days in culture. Higher magnification images (g, h, i) show normal cellular architecture at day 0 and day 3 with significant changes at day 5.

**Figure 5:**
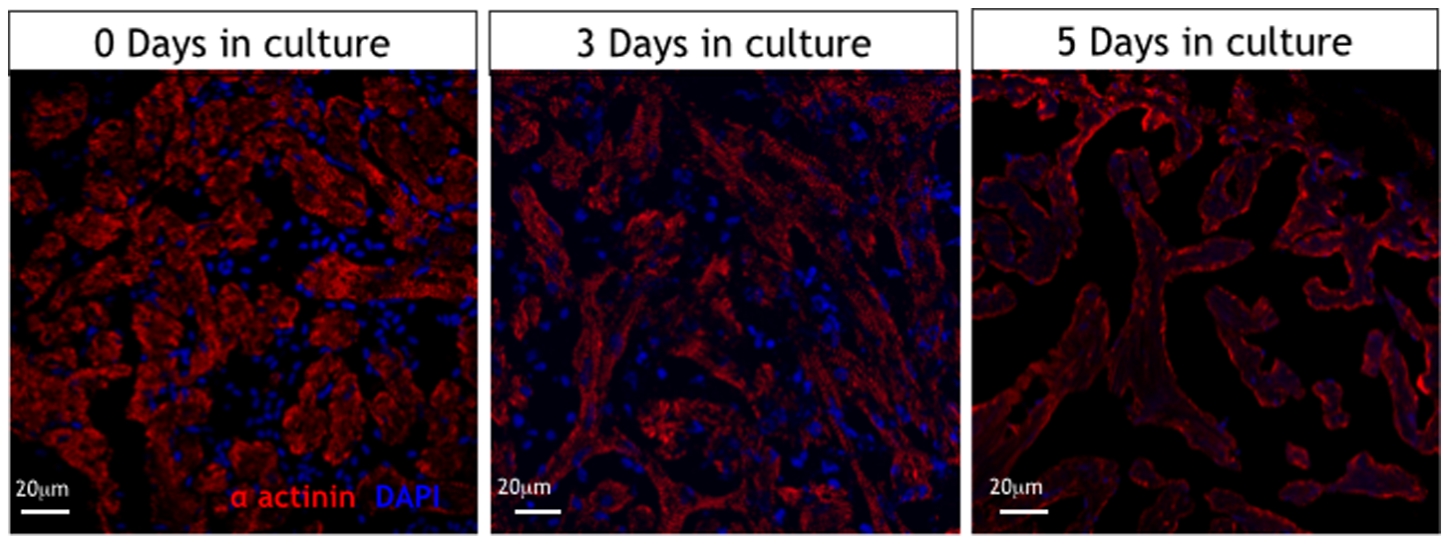
Immunohistology of isolated cultured hearts. Alpha-actinin immunostaining showing well preserved sarcomere patterns in cardiomyocytes of cultured hearts at day 0 and day 3 with loss of this typical pattern by day 5 in culture. (red is sarcomeric alpha-actinin (mouse, clone EA-53, Abcam), blue is DAPI (Serva Heidelberg)).

In the majority of hearts (>95%) we did not observe significant differences in the histological appearances of the ventricular myocardium on day 0 compared with hearts cultured for 3 days (Figure 4, d,e,g,h). Intact nuclei were clearly visible throughout sections from freshly excised hearts and hearts maintained in culture for up to 3 days. Associated cardiomyocytes retained their pale eosinophilic cytoplasm and cross striation architecture confirming the presence of viable cells with evidence of normal abundance of collagenous scaffold as revealed by Masson’s trichrome (blue staining pattern in Figures 4 (m,h,p,q) ). However, on day 5 there was evidence of pyknosis (nuclear condensation) and karryorhexis (nuclear fragmentation) with cardiomyocyte cell swelling and a loss of the normal cytoplasmic integrity. Degenerative changes appeared to develop first within outer layers of compact and spongy myocardium with the apex particularly affected. Immunohistology using alpha-actinin (figure 5) showed intact sarcomeric organization of the cultured zebrafish hearts on days 0 to 3 in culture. By day 5 there was a clear loss of the typical striation pattern, reduced numbers of nuclei staining clearly with DAPI and a change in the normal cardiomyocyte architecture.

## Discussion

This study has demonstrated that, under the specific experimental conditions described, adult zebrafish hearts can be excised whole, placed on a culture plate in standard medium in an unloaded state where they will maintain some degree of function and cellular integrity for up to 3 days. Beyond this time point a number of functional and histological features begin to appear that suggest a significant and progressive loss of tissue integrity and function. Remarkably, the response of heart rate and ventricle wall motion to beta-adrenergic stimulation remains intact for up to 3 days in culture suggesting that this signalling pathway remains intact and responsive despite a general decline in heart rate and function.

Millhouse *et* *al* (1971) showed that beating larval salamander hearts could be maintained for up to 6 months in culture [20] although these hearts showed major changes in sarcomeric organization accompanied by dedifferentiation of contractile machinery after such an extended period in culture. Standard histological assessment was not reported in this study and so other gross architectural changes in myocardial tissue similar to those observed in our zebrafish hearts were not reported. Isolated adult zebrafish hearts have been previously studied using a force transducer to measure twitch force generated by the beating heart [18]. While, these authors report the ability to maintain hearts for up to 4 hours the experimental setup did not allow longer term assessment and analysis of multiple hearts. Our study aimed to optimise culture conditions using multiple adult zebrafish hearts to assess the feasibility of using this technique for high throughput histological and functional studies.

The mean heart rate of isolated adult zebrafish hearts placed in culture-medium on the day of excision (day 0) was around 100 bpm which is lower than previously published values of *in* *vivo* heart rates for adult zebrafish at 28°C (120–130 bpm, [21]). Interestingly, the beta agonist isoproterenol restored the heart rate to more physiological levels suggesting the possibility that there may be background tonic adrenergic drive *in-vivo* maintaining heart rate at 120 to 130 bpm. Our hearts were maintained at 28°C but even a small reduction in temperature could have had a profound effect on heart rate in the cultured heart [18]. It is also possible that surgical excision of the heart caused a loss of innervation or hormonal drive in the adult zebrafish heart resulting in lower observed heart rate in culture. There was a clear response of heart rate to isoproterenol at day 0 which remained intact on days 2 and 3 before being lost on day 4 and 5. This suggests that catecholamine receptors and their downstream signalling pathways remain intact and functional for up to 3 days in culture despite a significant decline in contractile function during the same time period. Contractile function, assessed by movement of a portion of the ventricle wall close to the apex, also showed a significant response to isoproterenol for up to 3 days in culture and thereafter declined markedly with very little motion of the wall by day 5. Once again, this suggests that the beta adrenergic pathways remain intact in the ventricle myocardium throughout this time period and that the myocardium retains some degree of contractile reserve despite diminishing baseline function. Interestingly, the baseline velocity of contraction and relaxation declined significantly by day 1 in culture and thereafter remained low compared to day 0 with very little response to isoproterenol. These measures of contractile function are highly dependent on energy supply, particularly relaxation. The fact that zebrafish hearts in culture show this pattern of response suggests that metabolic changes may be a significant limiting factor in these preparations. Indeed, interpretation of the findings needs to acknowledge the fact that oxygen tension in the culture medium is uncertain and is unlikely to be physiological. A further limiting factor is that the hearts are haemodynamically unloaded and this may affect the measured contractile function and may also have affected the survival of the hearts in culture. Approaches to maintaining hearts in a more physiological state with loading using intra-ventricular balloons and with optimised oxygen tensions require to be explored in future experiments.

Over 3 days in culture the majority of hearts showed only mild changes in cardiomyocyte architecture and striation pattern. Nuclear DAPI staining indicated that cardiomyocytes maintained nuclear integrity at least until day 3 in culture. Immunofluorescence microscopy with antibodies to alpha-actinin revealed intact cross-striations within cardiomyocytes consistent with ongoing contraction and the ability of the heart to respond to isoproterenol. TUNEL staining revealed significant increases in apoptotic nuclei by day 3 which was sustained at day 5 suggesting a significant degree of programmed cell death between these time points. Between day 3 and 5 in culture there was a clear loss of normal cellular architecture, loss of nuclear shape and size and loss of contractile apparatus all of which coincide with the marked decline in heart rate and contractile function.

These observations suggest that the isolated cultured adult zebrafish heart could provide a model for assessing functional and histological changes possibly for up to 3 days although the appearance of increasing apoptosis at this stage may represent a limiting factor.

Maintenance in culture beyond this time point was associated with significant decline in heart rate, contractile function and loss of response to beta agonist stimulation. These features combined with the increase in programmed cell death at day 3 limit the utility of this model beyond this time point. Further studies are required to assess whether more optimal culture conditions could be developed to improve longevity of these preparations using, for example, hearts from younger fish, different oxygen tensions in the culture medium, electrical pacing, haemodynamic loading or microperfusion of the ventricle.

## Conclusion

The adult beating zebrafish heart can be maintained in culture in an unloaded state for up to 3 days while maintaining a high degree of histological and functional integrity. Combining these culture experiments with a high throughput histological approach could allow the assessment of short to medium term responses of *ex-vivo* beating hearts to various pharmacological, genetic and molecular manipulations.
